# Using a Nonparametric Multilevel Latent Markov Model to Evaluate Diagnostics for Trachoma

**DOI:** 10.1093/aje/kws345

**Published:** 2013-04-01

**Authors:** Artemis Koukounari, Irini Moustaki, Nicholas C. Grassly, Isobel M. Blake, María-Gloria Basáñez, Manoj Gambhir, David C. W. Mabey, Robin L. Bailey, Matthew J. Burton, Anthony W. Solomon, Christl A. Donnelly

**Keywords:** diagnosis, latent Markov model, multilevel, nonparametric model, trachoma

## Abstract

In disease control or elimination programs, diagnostics are essential for assessing the impact of interventions, refining treatment strategies, and minimizing the waste of scarce resources. Although high-performance tests are desirable, increased accuracy is frequently accompanied by a requirement for more elaborate infrastructure, which is often not feasible in the developing world. These challenges are pertinent to mapping, impact monitoring, and surveillance in trachoma elimination programs. To help inform rational design of diagnostics for trachoma elimination, we outline a nonparametric multilevel latent Markov modeling approach and apply it to 2 longitudinal cohort studies of trachoma-endemic communities in Tanzania (2000–2002) and The Gambia (2001–2002) to provide simultaneous inferences about the true population prevalence of *Chlamydia trachomatis* infection and disease and the sensitivity, specificity, and predictive values of 3 diagnostic tests for *C. trachomatis* infection. Estimates were obtained by using data collected before and after mass azithromycin administration. Such estimates are particularly important for trachoma because of the absence of a true “gold standard” diagnostic test for *C. trachomatis*. Estimated transition probabilities provide useful insights into key epidemiologic questions about the persistence of disease and the clearance of infection as well as the required frequency of surveillance in the postelimination setting.

An essential component of programs for the evaluation and improvement of global health is the availability of accurate diagnosis. Yet current diagnostics for many infectious diseases do not meet the needs of the developing world (1). Additionally, the lack of a diagnostic “gold standard” against which the accuracy of these tests would be evaluated compounds the problem. Trachoma, caused by ocular infection with the bacterium *Chlamydia trachomatis*, is the leading infectious cause of blindness in poor communities of developing countries (2) and is a disease with exactly these diagnostic challenges.

Although polymerase chain reaction (PCR)–based assays are considered to be very sensitive and specific for the diagnosis of *C. trachomatis* infection, they are currently unavailable in trachoma-endemic settings and too expensive to be used routinely as impact evaluation tools in large-scale treatment programs. Therefore, the methods currently used to evaluate the success of control strategies are clinical examinations to detect the presence of active disease (trachomatous inflammation, follicular (TF) and trachomatous inflammation, intense (TI)). The World Health Organization recommends annual mass drug administration with azithromycin wherever the district prevalence of TF in children aged 1–9 years is 10% or greater (3). However, the diagnostic performance of clinical examination is hindered by the frequent presence of active disease in the absence of infection and of infection in the absence of active disease (4–6). This is partly because clinical signs of trachoma may persist for many weeks after infection has been cleared (6–8) and also may result when infections other than *Chlamydia* cause trachoma-like inflammatory disease (9). Consequently, detection of active trachoma signs may not constitute the best tool to evaluate control interventions. Furthermore, policy makers require accurate surveillance of disease to decide when treatment aimed at eliminating infection can be discontinued and to detect reemergence in previously treated areas (10). Also of importance as implementation is rolled out is the question of whether and how the diagnostic performance of the available tests used at baseline may change with initial endemicity and after mass drug administration.

Latent variable modeling, that is statistical modeling that includes unobserved random variables that can alternatively be thought of as underlying parameters, has been used in medical research for the analysis of diagnostic accuracy and disease progression parameters in the absence of an acceptable gold standard (11, 12), as in the case of trachoma. For instance, Grassly et al. (13) used a multistate hidden Markov model to estimate the median duration of active disease and infection for different age groups by using a longitudinal data set followed up every 2 weeks over a 6-month period in The Gambia. Estimates were also obtained for the sensitivity and specificity of PCR or for clinical symptoms for infection, but without distinguishing between TF and TI. Hidden Markov models have been used to describe processes in which an individual moves through a series of states in continuous time, such as over the course of chronic and infectious diseases (14–18), most often by using a single diagnostic test. Through latent class analysis, 2 recent studies evaluated the performance of the trachoma diagnostics DNA-PCR, TF (i.e., detection of clinical symptoms of TF), and TI (i.e., detection of clinical symptoms of TI) (10) as well as DNA-PCR, RNA-PCR, and TF (19) by using cross-sectional data from pretreatment and posttreatment hyperendemic areas in Ethiopia, respectively. Latent class analysis allows the results of several diagnostic tests to be analyzed simultaneously, allowing for measurement error and using the information from all the diagnostic indicators to make a diagnosis for each individual in the study (20, 21).

In the current study, we developed latent Markov models (LMMs), which we applied to 2 longitudinal data sets from Tanzanian and Gambian communities with low baseline trachoma prevalence before and after mass administration of azithromycin. We then assessed the diagnostic accuracy of DNA-PCR, TF, and TI in the absence of a gold standard for baseline and follow-up time points. LMMs can be seen as an extension of latent class analysis for the analysis of longitudinal data, and they have been proposed for public health research in several studies (22–24). However, their application to the evaluation of diagnostic accuracy has not, to our knowledge, been previously investigated.

All models mentioned above—both hidden Markov models and LMMs (i.e., extensions of continuous and discrete Markov chains, respectively, that deal with measurement error) as well as latent class analysis—are mixture models. In the current study, we extended existing methodological research by fitting multilevel LMMs and by allowing some of the model parameters to differ across households. This approach is novel in that it takes into consideration the nested structure of the data and overcomes computational challenges associated with multilevel longitudinal mixture models; it allows for simultaneous evaluation of the 3 diagnostic tests with a test of measurement invariance as well as evaluation of the effect of a mass drug administration–based intervention on model-estimated infection and disease prevalence over time in the absence of a gold standard.

## MATERIALS AND METHODS

### Study areas, data collection, and analysis

Studies were conducted in East and West Africa (in the Kahe Mpya subvillage of the Rombo District, Tanzania and in the Upper Saloum District, The Gambia). The presence of ocular *C. trachomatis* infections at baseline and follow-up times was assessed through a DNA-PCR assay (Amplicor PCR; Roche Diagnostics Corp., Indianapolis, Indiana). Active disease was defined as the presence of TF and/or TI by using the World Health Organization's simplified grading system (25). Detailed demographic information was collected, including individual's age, sex, and household membership. Full descriptions of the study populations, structure of their communities, laboratory methods used, and summary statistics have been published elsewhere (7, 26–31). We fitted models to the principal reservoir of infection (27) (i.e., children aged <10 years) because trachoma control programs currently make mass drug administration decisions on the basis of the prevalence of active disease in children aged 1–9 years.

**Figure 1.**
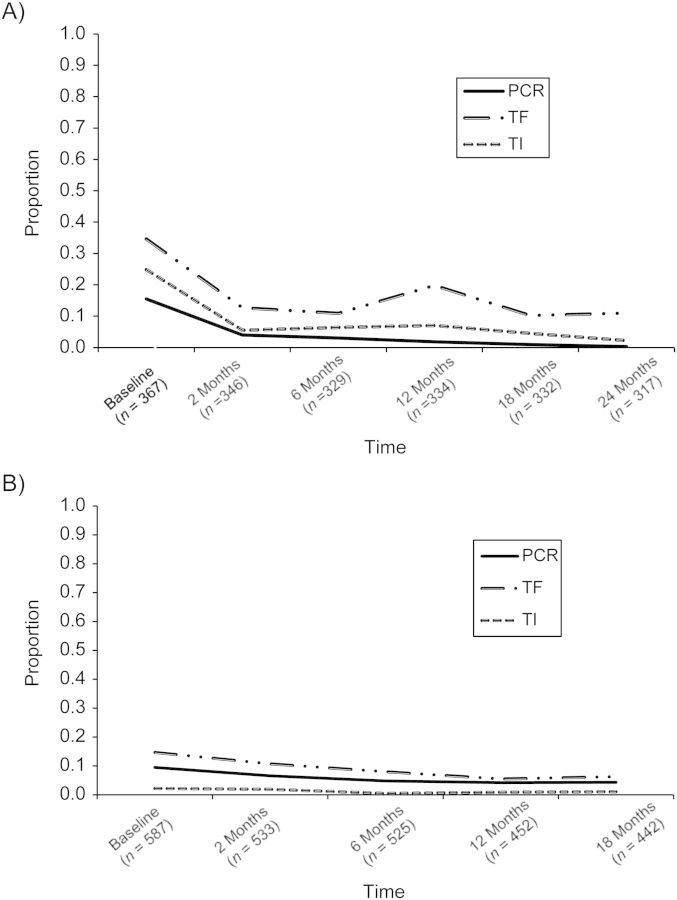
Descriptive (proportion positive) results according to the 3 diagnostic tests under investigation (DNA-PCR for *Chlamydia trachomatis* infection and TF and TI for signs of active trachoma disease) for children aged <10 years prior to and after mass azithromycin administration in A) Tanzania (2000–2002) and B) The Gambia (2001–2002). Numbers of children participating at each time point of the surveys are indicated in parentheses. PCR, polymerase chain reaction; TF, trachomatous inflammation, follicular; TI, trachomatous inflammation, intense.

For all subjects, follow-up examinations and eye swabbing were performed by the same examiner at 2, 6, 12, 18, and 24 months after mass drug administration in Tanzania (during 2000–2002) and at 2, 6, 12, and 18 months in The Gambia (during 2001–2002). In Tanzania, only 1 person had a positive swab at 24 months (Figure 1); therefore, statistical analysis was restricted to the first 5 time points for this data set. A single round of mass administration of azithromycin was offered following baseline data collection to all residents of communities participating in these studies. Because the work presented here involves only further statistical analyses of previously published, deidentified data obtained in the original studies (28–31) (which had been granted ethical approval), additional ethical approval was not required.

### Model specification for binary indicators

LMM consists of a structural model for the latent health states (analogous to the latent classes in latent class analysis) and a measurement model for the observed indicators (the 3 diagnostic tools: PCR, TI, and TF) conditional on latent health state.

Let *Y***_{*it*}_** = (*y_i1t_*, …, *y_iPt_*) be a response pattern for the *i*th individual at time *t* on *P* observed binary indicators (in this study the observed indicators are the 3 binary diagnostic tests, *P* = 3) with values 0 and 1 indicating negative and positive diagnostic test results, respectively.

We assume that for each subject, the true underlying health state at each discrete time point *t* (where *t* = 1, …, *T* and *T* = 5: baseline, 2, 6, 12, and 18 months) is explained by a latent categorical variable denoted by *C* with *J* latent health states.

The responses to the (*P* × *T*) *y* indicators are assumed to be independent conditional on the latent health state membership, which in our analysis implies that the results from the 3 diagnostics are assumed to be independent conditional on the true underlying health state both within and across time points. The latent categorical variable *C_i,t_* depends on *C_i,t_*_− 1_ but not on earlier latent categorical variables, known as the first-order Markov property. Under these assumptions, the probability of observing a particular response pattern *Y* for a randomly selected individual *i* is the following:
(1)
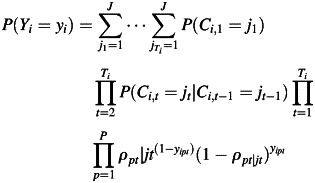

where
(2)
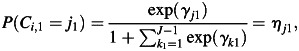

represents the baseline health state prevalence at the first time point (i.e., baseline), and
(3)
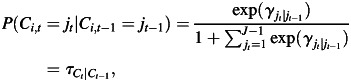

represents the probability of a transition to latent health state *j_t_* at time *t* conditional on membership in latent health state *j_t_*_− 1_ at time *t* − 1. 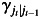 stands for the coefficients from multinomial logistic regressions for transitions between *t* − 1 and *t*.

Furthermore, the probability of a negative response to *p*th indicator at time *t* conditional on latent health state *j* at time *t* is given by
(4)
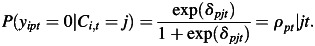



The ρ quantities are known in the latent variable modeling literature as the item response probabilities, and they show precisely how the indicators (i.e., the diagnostic tests) measure the latent health states. In the case where the latent health states were dichotomous (identified as “not infected” and “infected”) and 0 represented negative diagnostic test results, equation 4 would represent the diagnostic specificity for infection when *C_i,t_* is a “not infected” health state and 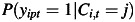 would represent the sensitivity for infection of the 3 diagnostics, respectively, when *C_i,t_* is an “infected” health state.

**Figure 2.**
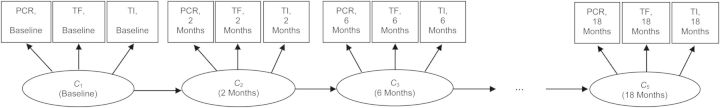
Latent Markov modeling path diagram. The variables in boxes represent the 3 observed categorical indicators of the latent categorical variables at each time point, *C_t_*. The 4 arrows between the circled variables indicate the regression model for the latent categorical variable at time point *t* on the latent categorical variable at time point *t* − 1. PCR, polymerase chain reaction; TF, trachomatous inflammation, follicular; TI, trachomatous inflammation, intense.

Depending on the number of latent health states (reflecting combinations of infected/not infected with diseased/not diseased), we derived estimates of sensitivities and specificities as well as positive and negative predictive values for all 3 diagnostic tools for ocular chlamydial infection on the basis of the estimates of η and ρ (equations 2 and 4) of relevant latent health states. For more technical details, see equations W6–W9 in the Web Appendix available at http://aje.oxfordjournals.org/. Figure 2 presents our LMM model in a path diagram.

Table 1 summarizes the notation for our proposed models.

**Table 1.** Latent Markov Model Notation

Symbol	Definition
*Structural model*	
*C*	Categorical latent variable at the individual level representing health (level 1)
η	Proportion of individuals belonging in a specific category of *C*/latent health state (or prevalence of latent health state)
γ	Coefficient for latent variable *C*
τ	Transition probability
*J*	Total number of latent health states
*j*	Index for latent health states of *C*
Cb^a^	New categorical latent variable at the household level (level 2) capturing the cluster variability in the distribution of the level-1 latent health states proportions
*Measurement model*	
*y*	Observed indicator (i.e., diagnostic test) of *C*
ρ	Item response probability
*i*	Index for individual
*T*	Total number of observed time points varies across individuals
*t*	Index for time
*p*	Index for diagnostic test
*P*	Total number of observed indicators
*n*	Sample size
δ	Coefficient relating *y* to *C*

^a^ See section, “Maximum likelihood estimation and numerical challenges” and Web Appendix for further technical details.

### Model selection

In mixture modeling, statistical theory determines the best model that combines goodness of fit and parsimony through minimum values for information criteria. In the current study, we explored these aspects among models that are biologically plausible for the epidemiological settings studied here (given the effect of mass drug administration on the natural history of trachoma). The inspection of information criteria informed our decisions (Web Table 1) and helped us explore the initial specifications of simple LMMs that did not account for household clustering of individuals. We made decisions with respect to the number of latent health states *J* required to obtain a good fit.

1. We then tested whether the transition probability matrix was stationary following treatment (i.e., 1 transition probability matrix between baseline and first follow-up and then another for subsequent transition periods vs. a separate transition probability matrix for each transition period). A global stationarity assumption (i.e., the equality of transition probability τ's across each of the transition points in LMMs) was not tested because this was not consistent with prior knowledge of mass drug administration impacts (28–30).
2. The hypothesis of measurement invariance of item response probabilities ρ's across time was also tested. In general, if the ρ's change over time, it is no longer as clear how to interpret the transition probabilities τ's because along with interpreting quantitative changes in latent health state membership, the meaning of the latent health states change over time.

Clustering of infection by household is a key characteristic of trachoma epidemiology (26). To allow for the nested structure of our 2 data sets (individuals within households), we expanded the selected model such that the categorical latent variables had random intercepts that varied between households. We used a discrete unspecified mixing distribution (i.e., nonparametric random-intercepts LMM) to examine if there was between-household variation in the prevalence of latent health states. We performed likelihood ratio tests comparing simple and multilevel LMMs. Further details of all models considered are given in the Web Appendix.

### Maximum likelihood estimation and numerical challenges

**Figure 3.**
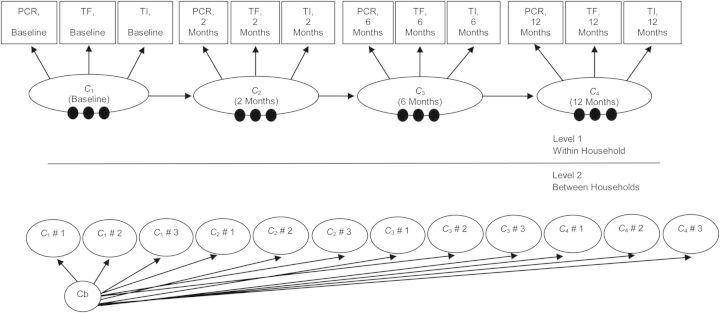
Nonparametric multilevel latent Markov modeling path diagram with *J* latent health states (*J* = 4). The within-household model (level 1) is similar to the model in Figure 2 with 3 observed categorical indicators and 4 measurement time points; the 3 single filled circles at the bottom of each of the 4 *C_t_* latent categorical variables represent the random means for the within-household latent health states (there are *J* − 1 random means). These random means are referred to as *C*_1_ # 1 …. *C*_4_ # 3 in the between-household model (level 2), and they vary across level 2 between households (cluster level) latent classes (labeled Cb in the above diagram). PCR, polymerase chain reaction; TF, trachomatous inflammation, follicular; TI, trachomatous inflammation, intense.

Maximum likelihood estimation of mixture modeling is nontrivial and time consuming; it requires an iterative algorithm because mixture models generally exhibit multiple local maxima of the likelihood, and so fitting procedures must be based on many starting values to ensure that a global maximum is found (32). In addition, maximum likelihood estimation of mixture models with random effects requires numerical integration, with each random effect with a non-0 level-2 variance contributing 1 dimension of integration (33, 34). Such problems have been circumvented for latent class analysis by the adoption of a nonparametric approach in which the computation burden is much less dependent on the number of random coefficients (35–37). We adopted this approach for LMM, with which we were able to obtain a nonparametric random-intercepts LMM for only the first 4 of the 5 time points of the study (see Figure 3). We fitted all models by using MPlus, version 6.1, software (38), which automatically uses appropriate iterative algorithms and several sets of random starting values to reduce the probability of obtaining a local solution. The method of estimation was full information maximum likelihood, in which we assumed that missing data were missing at random. This means we assumed that missingness in these data was related to the conditions of the data collection rather than the latent health states of individuals. In Tanzania, there were very high follow-up levels for the first 5 years, whereas in The Gambia the main reason for people not being seen at a particular follow-up appointment was travel outside the study area. We also performed χ^2^ tests for the missingness assumption. Such an approach allowed maximum use of data from individuals with incomplete (missing) data at the time points under study. Further technical details are given in the Web Appendix.

## RESULTS

We fitted measurement-invariant LMMs (because the meaning of the latent health states also remains constant over time for these models) with 2 and 4 transition probability matrices for 2, 3, or 4 latent health states (Web Table 1). For Tanzania, our results indicated that a model with 2 transition probability matrices (1 for the 0- to 2-month follow-up and 1 for each subsequent follow-up period) and 3 latent health states was the most appropriate. We considered a similar model with 4 latent health states, but the item response probabilities did not point to meaningful interpretation of the latent health states; thus, we chose the model with 3 latent health states. For The Gambia, our results indicated that the model with 4 latent health states and 2 transition probability matrices was the most appropriate. The pattern of item response probabilities across the diagnostic tests (shown in Table 2) clearly differentiates among the latent health states and appears to correspond to different infection and disease states. For these selected models, there was no evidence that the missing at random assumption was violated (*P* values for χ^2^ test for missing completely at random were 1.000). Nonparametric random effects on the latent health states were significant in both models (Web Table 2). The models failed to converge when data from the final observation were included in each country, which was most likely a result of the low prevalence of infection at this final follow-up time. We therefore present results based on the first 4 time points only. By examining the item response probabilities ρ in Table 2, we assigned epidemiologically relevant interpretations to the latent health states. The first latent health state, labeled as “not infected and not diseased,” was characterized by very high probabilities of yielding negative results in both countries in all 3 examined diagnostic tests. Then, for Tanzania, “not infected and diseased” and “infected and diseased” can be assigned to the complementary corresponding probabilities of the second and third latent health states, respectively. In The Gambia, where a 4-state LMM was supported, the additional state appeared to correspond to “infected and not diseased.”

**Table 2.** Item Response Probabilities^a^ ρ of Yielding Negative Results From a Nonparametric Multilevel Latent Markov Model for Each Diagnostic Test and Latent Health State in Tanzania (2000–2002) and The Gambia (2001–2002)

Latent Health State	Tanzania						The Gambia					
	PCR	95% CI	TF	95% CI	TI	95% CI	PCR	95% CI	TF	95% CI	TI	95% CI
I − /D −	0.990	0.972, 0.997	1.000^a^	1.000, 1.000	0.999	0.210, 1.000	0.992	0.982, 0.996	0.993	0.837, 1.000	0.999	0.992, 1.000
I + /D −							0.172	0.056, 0.421	0.812	0.655, 0.908	0.974	0.883, 0.995
I − /D +	0.983	0.818, 0.999	0.402	0.305, 0.508	0.707	0.634, 0.771	1.000	1.000, 1.000	0.083	0.000, 0.982	0.929	0.846, 0.969
I + /D +	0.222	0.055, 0.585	0.133	0.032, 0.415	0.357	0.182, 0.582	0.276	0.054, 0.717	0.000	0.000, 0.000	0.707	0.445, 0.879

Abbreviations: CI, confidence interval; I − /D − , not infected and not diseased; I + /D − , infected and not diseased; I − /D + , not infected and diseased; I + /D + , infected and diseased; PCR, polymerase chain reaction; TF, trachomatous inflammation, follicular; TI, trachomatous inflammation, intense.

^a^ These parameters were estimated close to 1 or 0, so to avoid numerical instability in the estimation algorithm, MPlus software (Muthén & Muthén, Los Angeles, California) fixed them automatically to 1 or 0, respectively. For this same reason, standard errors and confidence intervals are not provided.

**Figure 4.**
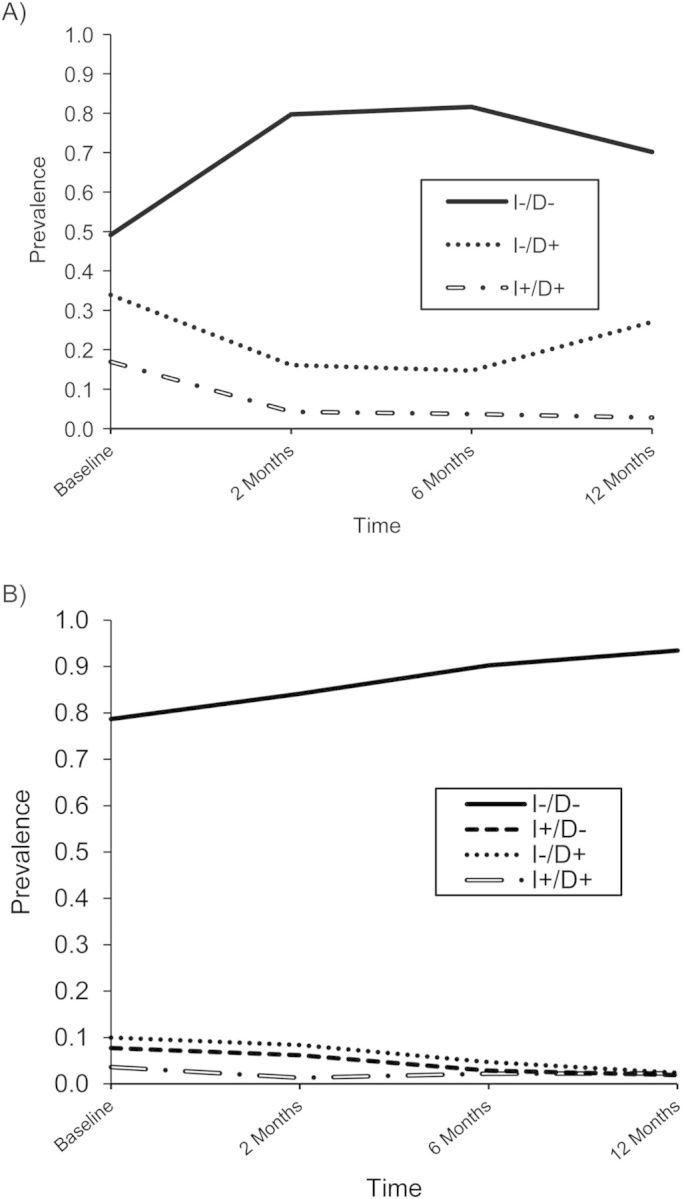
Estimates of prevalences η of latent health states over time as calculated from the nonparametric multilevel latent Markov model for A) Tanzania (2000–2002) and B) The Gambia (2001–2002). I − /D − , not infected and not diseased; I + /D − , infected and not diseased; I − /D + , not infected and diseased; I + /D + , infected and diseased.

Having assigned interpretations to the latent health states, we examined the estimated prevalences η of these latent health states over time (Figure 4). In both countries, the majority of children fell into the latent health state defined as “not infected and not diseased.” However, the proportion of those children who fell into this latent health state decreased in Tanzania after 6 months post–mass drug administration, whereas in The Gambia this proportion continued to increase over the study period. On the other hand, the proportion of children who fell into the latent health state, “not infected and diseased,” also increased in Tanzania after 6 months post–mass drug administration, whereas in The Gambia this proportion continued to decrease over the study period.

Furthermore, we examined the transitions between the latent health states over time by inspecting the elements of the transition probability matrices (Tables 3 and 4). Diagonal elements represent being in a latent health state at time *t,* conditional on being in that same latent health state at the previous time (*t* − 1). As expected, most of these elements were high in the nontreatment intervals. There was a higher probability of clearance of disease in uninfected individuals in The Gambia during both treatment and nontreatment intervals (τ = 0.557 and 0.790, respectively) compared with Tanzania (τ = 0.316 and 0.000, respectively).

**Table 3.** Transition Probability Results τ From the Nonparametric Multilevel Latent Markov Model for Tanzania, 2000–2002

Time, *t* – 1^a^	Treatment Interval (0–2 months), Time *t*			Nontreatment Intervals (2–6 months and 6–12 months), Time *t*		
	I − /D −	I − /D +	I + /D +	I − /D −	I − /D +	I + /D +
I − /D −	1.000	0.000	0.000	0.768	0.131	0.101
I − /D +	0.316	0.684	0.000	0.000	1.000	0.000
I + /D +	0.260	0.541	0.198	0.000	0.001	0.999

Abbreviations: I − /D − , not infected and not diseased; I + /D − , infected and not diseased; I − /D + , not infected and diseased; I + /D + , infected and diseased.

^a^ Time *t* – 1 represents the index for the immediately previous time point in the study.

**Table 4.** Transition Probability Results τ From the Nonparametric Multilevel Latent Markov Model for The Gambia, 2001–2002

Time *t* – 1^a^	Treatment Interval (0–2 months), Time *t*				Nontreatment Intervals (2–6 months and 6–12 months), Time *t*			
	I − /D −	I + /D −	I − /D+	I + /D +	I − /D −	I + /D −	I − /D +	I + /D +
I − /D −	0.980	0.000	0.016	0.004	0.985	0.000	0.015	0.000
I + /D −	0.721	0.000	0.279	0.000	0.000	1.000	0.000	0.000
I − /D +	0.557	0.000	0.443	0.000	0.790	0.040	0.170	0.000
I + /D +	0.000	0.000	1.000	0.000	0.434	0.000	0.566	0.000

Abbreviations: I − /D − , not infected and not diseased; I + /D − , infected and not diseased; I − /D + , not infected and diseased; I + /D + , infected and diseased.

^a^ Time *t* – 1 represents the index for the immediately previous time point in the study.

Finally, by combining estimates of η and ρ and averaging them over the 4 time points of study here (equations W6–W9 in the Web Appendix), we were able to evaluate the accuracy of all 3 diagnostic tests over time for ocular chlamydial infection for Tanzania and The Gambia (Table 5).

**Table 5.** Evaluation of Diagnostics for Detection of Infection From the Nonparametric Multilevel Latent Markov Model^a^ in Tanzania (2000–2002) and The Gambia (2001–2002)

	Tanzania						The Gambia					
	PCR, %	95% CI	TF, %	95% CI	TI, %	95% CI	PCR, %	95% CI	TF, %	95% CI	TI, %	95% CI
Sensitivity	77.8	41.5, 94.5^b^	86.7	58.5, 96.8^b^	64.3	41.8, 81.8^b^	79.0	58.6, 99.4	48.6	6.3, 90.8	12.4	0, 27^c^
Specificity	98.8	98.1, 99.6	85.8	73.6, 96.3	92.5	87.0, 98.1	99.2	98.9, 99.5	93.0	79.7, 100	99.4	98.2, 100
Positive predictive value	76.8	47.5, 100^c^	27.3	0, 64.6^c^	35.4	0, 78.6^c^	84.8	65.4, 100	31.3	0, 73.7^c^	55.4	0, 100^d^
Negative predictive value	98.3	96.3, 100	98.7	97.2, 100	97.1	94.4, 99.7	98.5	96.0, 100	95.8	85.4, 100^c^	93.8	82.4, 100^c^

Abbreviations: CI, confidence interval; PCR, polymerase chain reaction; TF, trachomatous inflammation, follicular; TI, trachomatous inflammation, intense.

^a^ The δ method has been used to approximate the standard errors for these functions of parameters as explained in the Web Appendix.

^b^ Because in the Tanzania analysis there is an I + /D + latent health state but no I + /D − latent health state, the sensitivity estimates and their confidence limits are obtained directly from the item response probabilities and their confidence limits displayed in Table 2 by using the equation that sensitivity equals 1 minus the item response probability for I + /D + .

^c^ Because the normal approximation (i.e., estimate plus or minus 1.96 times the estimated standard error) does not take into account intrinsic constraints on parameter values, confidence intervals estimated in this way could extend beyond 0% or 100%. In these cases, we have used 0% as the minimum or 100% as the maximum.

^d^ In this case, the approximate standard error was so large that the normal approximation to the 95% confidence interval spanned the range from 0% to 100%. Clearly, there is little information in the data set about this positive predictive value.

## DISCUSSION

We applied nonparametric multilevel LMMs to 2 longitudinal data sets from Tanzanian and Gambian communities with low baseline trachoma prevalence before and after a round of mass azithromycin administration. This latent variable modeling approach yielded an average assessment of the diagnostic accuracy of PCR, TF, and TI for the detection of *C. trachomatis* infection in the absence of a gold standard.

We believe that our findings have important implications for monitoring and evaluation of trachoma control programs. For instance, TF is the measure on which mass drug administration decisions are often based.

The sensitivity and positive predictive value of clinical examination for infection were estimated to be very low in The Gambia, whereas the sensitivity of TI and positive predictive value of clinical examination (TF and TI) were very low in Tanzania, which is problematic; if treatment decisions are based solely on the simplified grading system, it could potentially lead to the decision to continue the annual mass administration of antichlamydial antibiotics for years after *C. trachomatis* has been eliminated from recipient communities (39). TF had higher sensitivity but lower positive predictive value and specificity for infection than did TI. The sensitivity for infection of DNA-PCR was estimated to be 77.8% (95% confidence interval, 41.5%, 94.5%) in Tanzania and 79.0% (95% confidence interval, 58.6%, 99.4%) in The Gambia, which is comparable with estimates in Ethiopia (19, 40). A model with variable sensitivity and specificity of the diagnostic tests (varying item response probabilities) over time was not supported by the data in The Gambia (Web Table 1), although this may be the result of low power given the low infection prevalence after treatment in this community. In Tanzania, there was support for variable performance of the diagnostic tests over time (Web Table 1). This complicates the interpretation of the latent health states; therefore, in this analysis we focused on the model with fixed item response probabilities over time. However, further work should explore the significance of this variation over time; because a temporal relationship between infection and clinical disease will be driving the diagnostic performance results, it is unlikely that TF would have the same predictive value for infection at 2 or even 6 months after mass drug administration as it would in treatment-naïve populations.

Our nonparametric multilevel modeling approach allowed the latent health state prevalences to vary between households with the advantage of less strong distributional assumptions and computational burden for the random effects at the household level (Web Appendix). Maximum likelihood estimation of a parametric multilevel LMM (where a common factor would model the random means and their associated covariances) would require 4 dimensions of integration, resulting in large computational complexity (32, 35, 36).

These flexible nonparametric multilevel LMMs facilitated evaluation of the effect of a mass drug administration–based intervention on the prevalence of infection and disease over time. This is particularly important because the use of mass drug administration for trachoma control would ideally be based on the true prevalence of *C. trachomatis* infection (41). Such estimates may also shed light on the relationship between the prevalence of clinical activity and chlamydial infections for other comparable endemic settings. One of the novelties of our methodology is that such quantities were estimated by using latent variables, adjusting for the measurement error contained in the diagnostic tests studied. LMMs require only that there is a certain degree of correlation among the different diagnostic tests; TF and TI convey different information than does the PCR, and this is reflected here in the formation of the latent health states. We recognize though that our results depend upon the assumption of conditional independence.

On the basis of the latent health state prevalences at each time point alone, it is impossible to quantify how children move between these latent health states over time. The transition probability matrices (Tables 3 and 4) provide parsimonious yet detailed insights into key epidemiologic differences regarding the persistence of disease and the clearance of infection in the 2 studies. For instance, 1 of the key findings was the persistence of disease in Tanzania between 2 and 12 months, suggesting that tetracycline given to TF cases after baseline had little effect. Furthermore, the occurrence of disease resembling “active trachoma” in noninfected individuals living in communities that are or have recently been trachoma endemic is well documented (13, 42) and is the subject of ongoing research. The null or very low transition probabilities from “not infected and not diseased” to “infected and not diseased” might suggest a lack of field and laboratory cross-contamination of samples. Also, within the nontreatment interval, perhaps the transition from “infected and not diseased” to “not infected and not diseased” would be high if there were contamination in the samples between the different time points. However, we believe that contamination would be most probable at single time points among samples rather than between time points; thus, such interpretations should be made cautiously here. In addition, because of the designs of the longitudinal studies (specifically the sampling times of measurements performed), we recognize that these transition probabilities might well underestimate the true change between these latent health states over time (43). Because the time points are unevenly spaced, we could have fitted transition hazards rather than transition probabilities (in other words, hidden Markov models), but then only 1 diagnostic test would be evaluated because it is not computationally tractable to fit such models to multiple diagnostic tests simultaneously. Our fitted models have a separate transition probability matrix for the first time period (0–2 months, in which mass drug administration took place) and for subsequent transition periods. Thus, we have assumed the same transition probability in 4- and 6-month periods. We believe that this approximation is not crucial for our final inferences. However, in cases of less evenly spaced time points when simultaneous evaluation of multiple diagnostic tests with measurement error is not of primary interest, hidden Markov models might be more appropriate as a method of analysis. If there were more frequent evenly spaced measurements after mass drug administration, a more accurate picture of the posttreatment natural history of trachoma might emerge. We propose that those designing longitudinal trachoma studies to inform control programs consider the measurement frequency carefully where resourcing could allow for more frequent follow-up.

In conclusion, this study has demonstrated that nonparametric multilevel LMMs can be used for the evaluation of diagnostic tools for trachoma in the absence of a diagnostic gold standard, even while allowing for the inherent hierarchical structure of these data: namely, repeated observations of children within households. We recommend that these methods be applied in settings with moderate and high baseline trachoma prevalence where longitudinal data can be generated before and after mass drug administration. Such research will aid trachoma program managers in their efforts to identify and characterize useful measures to evaluate control interventions. More generally, we recommend that LMMs be used for the evaluation of diagnostic tests of other diseases without gold standard diagnostic tools whenever longitudinal data are available, because such methodology has greater statistical power than does latent class analysis. Because LMMs use the additional information provided by repeated measurements, they might enable detection of additional latent health states or latent classes than if a latent class analysis was used at each time point. Furthermore, LMMs permit questions about changes in true health states and test the measurement invariance hypothesis of the diagnostic tests of interest over time, making them very useful tools for control program monitoring and evaluation research.

## Supplementary Material

Web Material
