# Supplementary material for: Using a Nonparametric Multilevel Latent Markov Model to Evaluate Diagnostics for Trachoma
Source: Am J Epidemiol. 2013 Apr 1;177(9):913–22. doi: 10.1093/aje/kws345 (PMC3639724; doi:10.1093/aje/kws345)
Supplement: Web Material [file supp_177_9_913__index.html]

Using a Nonparametric Multilevel Latent Markov Model to Evaluate Diagnostics for Trachoma — Web Material 

# Using a Nonparametric Multilevel Latent Markov Model to Evaluate Diagnostics for Trachoma

## Web Material

**Files in this Data Supplement:**

- Web Material
