# Supplementary material for: Using a Nonparametric Multilevel Latent Markov Model to Evaluate Diagnostics for Trachoma
Source: Am J Epidemiol. 2013 Apr 1;177(9):913–22. doi: 10.1093/aje/kws345 (PMC3639724; doi:10.1093/aje/kws345)
Supplement: Web Material [file supp_kws345_kws345_Web_Material.pdf]

WEB MATERIAL

Nonparametric multilevel LMM

LMM assumes that subjects (here children) are independent. However, in our study children are nested within households and therefore the independence assumption is not fulfilled.

Multilevel LMMs account for the hierarchical structure of the data by allowing the latent health states intercepts to vary across households. The random intercepts then allow for the probability of belonging to a particular latent health state $P(C_{i,t} = j_t)$ to vary across households. Covariates or contextual effects (household characteristics) can then be added as level-2 covariates to explain the variation in those probabilities.

More specifically, in our study, the level-1 LMM is defined by four latent health states at each time point and therefore a multinomial logistic regression model is used with J-1 random intercepts at each time point where J is equal to the number of latent health states (here four or three). One latent state is taken as a reference category (MPLUS uses the last latent health state as the reference category) and therefore the introduction of the random intercepts allows the log-odds of belonging to a specific latent health state at each time point to vary across households. We follow here the nonparametric approach discussed by Henry and Muthen 2010 in which a second latent class model is specified at level 2; in this model a new between-level (level-2) categorical latent variable is denoted by *Cb.* A small number of level-2 latent classes capture the level-2 variability in the distribution of the level-1 latent health state membership probabilities. In this approach the normal distribution that is usually assumed for the random error of the intercept is replaced with the assumption of a multinomial distribution. Through this approach, clusters (i.e. households) are classified into a small number of types, rather than be placed on a

continuous scale (this would be the case if for instance household level random effects were considered as drawn from a normal distribution). This yields a nonparametric multilevel LMM in which there are not only latent categorical variables C_i with J latent health states of level-1 units but also latent classes of level-2 units sharing the same parameter values (i.e. the random means; that is the log-odds of membership in a particular level-1 latent health state).

The probability now that individual i in level-2 unit b is a member of latent health state j at time t is a conditional probability and is given by:

$$P(C_{i,b,t} = j \mid Cb_b = m) = \frac{\exp(\gamma_{jtm})}{\sum_{k=1}^{J} \exp(\gamma_{ktm})} \qquad \textbf{(W.1)}$$

where $\gamma_{jtm} = \gamma_{jt} + u_{tm}$, and γ_{jt} is the linear regression intercept for the log-odds of belonging to the j latent health state rather than the last latent health state and u_{tm} is the household random effect which comes from a discrete mixture distribution with m representing a specific mixture and capturing the between households variation in the log-odds.

Evaluation of the diagnostic accuracy of Polymerase Chain Reaction (PCR) , Trachomatous Inflammation-Follicular (TF), and Trachomatous Inflammation-Intense (TI) for *C. trachomatis* infection (technical details for derivation of measures in Table 5)

In general, sensitivity (Sens) is the probability that an individual who is truly positive (denoted *TP*) has a positive screening result (denoted +). Specificity (Spec) is the probability that an individual who is truly negative (denoted TN) has a negative screening result (denoted -). The positive predictive value (PPV) is the probability that an individual with a positive screening result is truly positive. The negative predictive value (NPV) is the probability that an individual with a negative screening result is truly negative.

Therefore,

$$Sens = \frac{P(+ \cap TP)}{P(TP)}$$

(W.2)

where $P(+ \cap TP)$ is the joint probability of having a positive screening result and being truly positive and $P(TP)$ is the probability of a randomly chosen member of the study population being screened to be truly positive.

$$Spec = \frac{P(- \cap TN)}{P(TN)}$$

(W.3)

Similarly $\Pr(-\cap TN)$ is the joint probability of having a negative screening result and being truly negative and $\Pr(TN)$ is the probability of a randomly chosen member of the population being screened to be truly negative.

$$PPV = \frac{Sens \times P(TP)}{Sens \times P(TP) + (1 - Spec) \times P(TN)}$$

(W.4)

$$NPV = \frac{Spec \times P(TN)}{Spec \times P(TN) + (1 - Sens) \times P(TP)}$$

(W.5)

For the current study we consider from the LMMs, the four latent health states as identified in Table 2 as follows:

1) Not Infected & Non Diseased (denoted I-,D-)

2) Infected & Non Diseased: (denoted I+,D-)

3) Not Infected & Diseased: (denoted I-,D+)

4) Infected & Diseased: (denoted I+,D+)

Therefore for each time point and each diagnostic test, based on the formulas above we calculate as follows,

Sensitivity$_{pt}$ for Infection =

$$\frac{P_p(+\mid \mathrm{I+,D-})\times \mathrm{P_t(I+,D-)} + P_p(+\mid \mathrm{I+,D+})\times \mathrm{P_t(I+,D+)}}{\mathrm{P_t(I+,D-)} + \mathrm{P_t(I+,D+)}},\ p=1,2,3;t=1,2,3,4 \tag{W.6}$$

Specificity$_{pt}$ for Infection =

$$\frac{P_p(-\mid \mathrm{I-,D+})\times \mathrm{P_t(I-,D+)} + P_p(-\mid \mathrm{I-,D-})\times \mathrm{P_t(I-,D-)}}{\mathrm{P_t(I-,D+)} + \mathrm{P_t(I-,D-)}},\ p=1,2,3;t=1,2,3,4 \tag{W.7}$$

PPV$_{pt}$ for Infection =

$$\frac{P_p(+\mid \mathrm{I+,D-})\times \mathrm{P_t(I+,D-)} + P_p(+\mid \mathrm{I+,D+})\times \mathrm{P_t(I+,D+)}}{P_p(+\mid \mathrm{I+,D-})\times \mathrm{P_t(I+,D-)} + P_p(+\mid \mathrm{I+,D+})\times \mathrm{P_t(I+,D+)} + P_p(+\mid \mathrm{I-,D+})\times \mathrm{P_t(I-,D+)} + P_p(+\mid \mathrm{I-,D-})\times \mathrm{P_t(I-,D-)}},\ p=1,2,3;t=1,2,3,4 \tag{W.8}$$

NPV$_{pt}$ for Infection=

$$\frac{P_p(-\mid \mathrm{I-,D+})\times \mathrm{P_t(I-,D+)} + P_p(-\mid \mathrm{I-,D-})\times \mathrm{P_t(I-,D-)}}{P_p(-\mid \mathrm{I+,D-})\times \mathrm{P_t(I+,D-)} + P_p(-\mid \mathrm{I+,D+})\times \mathrm{P_t(I+,D+)} + P_p(-\mid \mathrm{I-,D+})\times \mathrm{P_t(I-,D+)} + P_p(-\mid \mathrm{I-,D-})\times \mathrm{P_t(I-,D-)}},\ p=1,2,3;t=1,2,3,4 \tag{W.9}$$

where,

$P_p(+\mid \mathrm{I+},\mathrm{D-})$, $P_p(+\mid \mathrm{I+},\mathrm{D+})$, $P_p(-\mid \mathrm{I-},\mathrm{D+})$, $P_p(-\mid \mathrm{I-},\mathrm{D-})$, in (S.6-S.9) are conditional probabilities and represent the item response probabilities ρ for the p diagnostic tests as estimated from the nonparametric multilevel LMMs contained in Table 2 in the main article.

$\mathrm{P_t}(\mathrm{I+},\mathrm{D-})$, $\mathrm{P_t}(\mathrm{I+},\mathrm{D+})$, $\mathrm{P_t}(\mathrm{I-},\mathrm{D+})$, $\mathrm{P_t}(\mathrm{I-},\mathrm{D-})$ in (W.6-W.9), are the prevalences η of the latent health states J. The subscript t denotes that these quantities change at each time point of the studies as estimated from the nonparametric multilevel LMMs (these are illustrated in Figure 4). After these calculations at each time point we take the average of each of these quantities.

Computation of approximated standard errors for the measures of Sensitivity (W.6), Specificity (W.7), PPV (W.8) and NPV (W.9).

We employ here the Delta method for computing approximated standard errors for the functions of those parameters given in W.6-W.9. For the prevalence estimates only we used the conservative standard errors from the fixed effects models since it was not feasible to acquire those from the random effects models from the MPLUS output.

We provide below the first derivatives of the measures given in W.6-W.9 with respect to the individual parameters they are functions of.

First derivatives of Sensitivity

w.r.t $P_p(+\mid \mathrm{I+,D-})$: $S_{pt1} = \dfrac{\mathrm{P_t(I+,D-)}}{[\mathrm{P_t(I+,D-)} + \mathrm{P_t(I+,D+)}]}, \; p = 1,2,3$

w.r.t. $P_p(+\mid \mathrm{I+,D+})$: $S_{pt2} = \dfrac{\mathrm{P_t(I+,D+)}}{[\mathrm{P_t(I+,D-)} + \mathrm{P_t(I+,D+)}]}, \; p = 1,2,3$

w.r.t. $\mathrm{P_t(I+,D-)}$: $S_{pt3} = \dfrac{[P_p(+\mid \mathrm{I+,D-}) - P_p(+\mid \mathrm{I+,D+})] \times \mathrm{P_t(I+,D+)}}{[\mathrm{P_t(I+,D-)} + \mathrm{P_t(I+,D+)}]^2}, t = 1,2,3,4; \; p = 1,2,3$

w.r.t. $\mathrm{P_t(I+,D+)}$: $S_{pt4} = \dfrac{[P_p(+\mid \mathrm{I+,D+}) - P_p(+\mid \mathrm{I+,D-})] \times \mathrm{P_t(I+,D-)}}{[\mathrm{P_t(I+,D-)} + \mathrm{P_t(I+,D+)}]^2}, t = 1,2,3,4; \; p = 1,2,3$

First derivatives of Specificity

w.r.t. $P_p(-\mid \mathrm{I-,D-})$: $\dfrac{\mathrm{P_t(I-,D-)}}{[\mathrm{P_t(I-,D+)} + \mathrm{P_t(I-,D-)}]}, \; p = 1,2,3$

w.r.t. $P_p(-\mid \mathrm{I-,D+})$: $\dfrac{\mathrm{P_t(I-,D+)}}{[\mathrm{P_t(I-,D+)} + \mathrm{P_t(I-,D-)}]}, \; p = 1,2,3$

w.r.t. $P_t(\mathrm{I-,D-})$: $\dfrac{[P_p(-\mid \mathrm{I-,D+}) - P_p(-\mid \mathrm{I-,D-})] \times \mathrm{P_t(I-,D+)}}{[\mathrm{P_t(I-,D+)} + \mathrm{P_t(I-,D-)}]^2}, t = 1,2,3,4; \; p = 1,2,3$

w.r.t. $P_t(\mathrm{I-,D+})$: $\dfrac{[P_p(-\mid \mathrm{I-,D+}) - P_p(-\mid \mathrm{I-,D-})] \times \mathrm{P_t(I-,D-)}}{[\mathrm{P_t(I-,D+)} + \mathrm{P_t(I-,D-)}]^2}, t = 1,2,3,4; \; p = 1,2,3$

First derivatives of PPV

w.r.t. $P_p(+ | \mathrm{I+}, \mathrm{D-})$:

$$\frac{\mathrm{P_t}(\mathrm{I+},\mathrm{D-}) \times [P_p(+|\mathrm{I-},\mathrm{D-}) \times \mathrm{P_t}(\mathrm{I-},\mathrm{D-}) + P_p(+|\mathrm{I-},\mathrm{D+}) \times \mathrm{P_t}(\mathrm{I-},\mathrm{D+})]}{[P_p(+|\mathrm{I+},\mathrm{D-}) \times \mathrm{P_t}(\mathrm{I+},\mathrm{D-}) + P_p(+|\mathrm{I+},\mathrm{D+}) \times \mathrm{P_t}(\mathrm{I+},\mathrm{D+}) + P_p(+|\mathrm{I-},\mathrm{D+}) \times \mathrm{P_t}(\mathrm{I-},\mathrm{D+}) + P_p(+|\mathrm{I-},\mathrm{D-}) \times \mathrm{P_t}(\mathrm{I-},\mathrm{D-})]^2}, \; p = 1,2,3$$

w.r.t. $P_p(+ | \mathrm{I+}, \mathrm{D+})$:

$$\frac{\mathrm{P_t}(\mathrm{I+},\mathrm{D+}) \times [P_p(+|\mathrm{I-},\mathrm{D-}) \times \mathrm{P_t}(\mathrm{I-},\mathrm{D-}) + P_p(+|\mathrm{I-},\mathrm{D+}) \times \mathrm{P_t}(\mathrm{I-},\mathrm{D+})]}{[P_p(+|\mathrm{I+},\mathrm{D-}) \times \mathrm{P_t}(\mathrm{I+},\mathrm{D-}) + P_p(+|\mathrm{I+},\mathrm{D+}) \times \mathrm{P_t}(\mathrm{I+},\mathrm{D+}) + P_p(+|\mathrm{I-},\mathrm{D+}) \times \mathrm{P_t}(\mathrm{I-},\mathrm{D+}) + P_p(+|\mathrm{I-},\mathrm{D-}) \times \mathrm{P_t}(\mathrm{I-},\mathrm{D-})]^2}, \; p = 1,2,3$$

w.r.t. $P_p(+ | \mathrm{I-}, \mathrm{D-})$:

$$\frac{(-1) \times \mathrm{P_t}(\mathrm{I-},\mathrm{D-}) \times [P_p(+|\mathrm{I+},\mathrm{D-}) \times \mathrm{P_t}(\mathrm{I+},\mathrm{D-}) + P_p(+|\mathrm{I+},\mathrm{D+}) \times \mathrm{P_t}(\mathrm{I+},\mathrm{D+})]}{[P_p(+|\mathrm{I+},\mathrm{D-}) \times \mathrm{P_t}(\mathrm{I+},\mathrm{D-}) + P_p(+|\mathrm{I+},\mathrm{D+}) \times \mathrm{P_t}(\mathrm{I+},\mathrm{D+}) + P_p(+|\mathrm{I-},\mathrm{D+}) \times \mathrm{P_t}(\mathrm{I-},\mathrm{D+}) + P_p(+|\mathrm{I-},\mathrm{D-}) \times \mathrm{P_t}(\mathrm{I-},\mathrm{D-})]^2}, \; p = 1,2,3$$

w.r.t. $P_p(+ | \mathrm{I-}, \mathrm{D+})$:

$$\frac{(-1) \times \mathrm{P_t}(\mathrm{I-},\mathrm{D+}) \times [P_p(+|\mathrm{I+},\mathrm{D-}) \times \mathrm{P_t}(\mathrm{I+},\mathrm{D-}) + P_p(+|\mathrm{I+},\mathrm{D+}) \times \mathrm{P_t}(\mathrm{I+},\mathrm{D+})]}{[P_p(+|\mathrm{I+},\mathrm{D-}) \times \mathrm{P_t}(\mathrm{I+},\mathrm{D-}) + P_p(+|\mathrm{I+},\mathrm{D+}) \times \mathrm{P_t}(\mathrm{I+},\mathrm{D+}) + P_p(+|\mathrm{I-},\mathrm{D+}) \times \mathrm{P_t}(\mathrm{I-},\mathrm{D+}) + P_p(+|\mathrm{I-},\mathrm{D-}) \times \mathrm{P_t}(\mathrm{I-},\mathrm{D-})]^2}, \; p = 1,2,3$$

w.r.t. $P_t(\mathrm{I+}, \mathrm{D-})$:

$$\frac{P_p(+\mid \mathrm{I+,D-})\times[P_p(+\mid \mathrm{I-,D-})\times \mathrm{P_t}(\mathrm{I-,D-})+P_p(+\mid \mathrm{I-,D+})\times \mathrm{P_t}(\mathrm{I-,D+})]}{[P_p(+\mid \mathrm{I+,D-})\times \mathrm{P_t}(\mathrm{I+,D-})+P_p(+\mid \mathrm{I+,D+})\times \mathrm{P_t}(\mathrm{I+,D+})+P_p(+\mid \mathrm{I-,D+})\times \mathrm{P_t}(\mathrm{I-,D+})+P_p(+\mid \mathrm{I-,D-})\times \mathrm{P_t}(\mathrm{I-,D-})]^2}, t=1,2,3,4,\ p=1,2,3$$

w.r.t. $P_t(\mathrm{I+,D+})$:

$$\frac{P_p(+\mid \mathrm{I+,D+})\times[P_p(+\mid \mathrm{I-,D-})\times \mathrm{P_t}(\mathrm{I-,D-})+P_p(+\mid \mathrm{I-,D+})\times \mathrm{P_t}(\mathrm{I-,D+})]}{[P_p(+\mid \mathrm{I+,D-})\times \mathrm{P_t}(\mathrm{I+,D-})+P_p(+\mid \mathrm{I+,D+})\times \mathrm{P_t}(\mathrm{I+,D+})+P_p(+\mid \mathrm{I-,D+})\times \mathrm{P_t}(\mathrm{I-,D+})+P_p(+\mid \mathrm{I-,D-})\times \mathrm{P_t}(\mathrm{I-,D-})]^2}, t=1,2,3,4,\ p=1,2,3$$

w.r.t. $P_t(\mathrm{I-,D-})$:

$$\frac{(\text{-}1)\times P_p(+\mid \mathrm{I-,D-})\times[P_p(+\mid \mathrm{I+,D-})\times \mathrm{P_t}(\mathrm{I+,D-})+P_p(+\mid \mathrm{I+,D+})\times \mathrm{P_t}(\mathrm{I+,D+})]}{[P_p(+\mid \mathrm{I+,D-})\times \mathrm{P_t}(\mathrm{I+,D-})+P_p(+\mid \mathrm{I+,D+})\times \mathrm{P_t}(\mathrm{I+,D+})+P_p(+\mid \mathrm{I-,D+})\times \mathrm{P_t}(\mathrm{I-,D+})+P_p(+\mid \mathrm{I-,D-})\times \mathrm{P_t}(\mathrm{I-,D-})]^2}, t=1,2,3,4;\ p=1,2,3$$

w.r.t. $P_t(\mathrm{I-,D+})$:

$$\frac{(\text{-}1)\times P_p(+\mid \mathrm{I-,D+})\times[P_p(+\mid \mathrm{I+,D-})\times \mathrm{P_t}(\mathrm{I+,D-})+P_p(+\mid \mathrm{I+,D+})\times \mathrm{P_t}(\mathrm{I+,D+})]}{[P_p(+\mid \mathrm{I+,D-})\times \mathrm{P_t}(\mathrm{I+,D-})+P_p(+\mid \mathrm{I+,D+})\times \mathrm{P_t}(\mathrm{I+,D+})+P_p(+\mid \mathrm{I-,D+})\times \mathrm{P_t}(\mathrm{I-,D+})+P_p(+\mid \mathrm{I-,D-})\times \mathrm{P_t}(\mathrm{I-,D-})]^2}, t=1,2,3,4;\ p=1,2,3$$

First derivatives of NPV

w.r.t. $P_p(-\mid \mathrm{I+,D-})$:

$$\frac{(-1)\times \mathrm{P_t}(\mathrm{I+,D-})\times[P_p(-\,|\,\mathrm{I-,D-})\times \mathrm{P_t}(\mathrm{I-,D-})+P_p(-\,|\,\mathrm{I-,D+})\times \mathrm{P_t}(\mathrm{I-,D+})]}{[P_p(-\,|\,\mathrm{I+,D-})\times \mathrm{P_t}(\mathrm{I+,D-})+P_p(-\,|\,\mathrm{I+,D+})\times \mathrm{P_t}(\mathrm{I+,D+})+P_p(-\,|\,\mathrm{I-,D+})\times \mathrm{P_t}(\mathrm{I-,D+})+P_p(-\,|\,\mathrm{I-,D-})\times \mathrm{P_t}(\mathrm{I-,D-})]^2},\ p=1,2,3$$

w.r.t. $P_p(-\,|\,\mathrm{I+,D+})$:

$$\frac{(-1)\times \mathrm{P_t}(\mathrm{I+,D+})\times[P_p(-\,|\,\mathrm{I-,D-})\times \mathrm{P_t}(\mathrm{I-,D-})+P_p(-\,|\,\mathrm{I-,D+})\times \mathrm{P_t}(\mathrm{I-,D+})]}{[P_p(-\,|\,\mathrm{I+,D-})\times \mathrm{P_t}(\mathrm{I+,D-})+P_p(-\,|\,\mathrm{I+,D+})\times \mathrm{P_t}(\mathrm{I+,D+})+P_p(-\,|\,\mathrm{I-,D+})\times \mathrm{P_t}(\mathrm{I-,D+})+P_p(-\,|\,\mathrm{I-,D-})\times \mathrm{P_t}(\mathrm{I-,D-})]^2},\ p=1,2,3$$

w.r.t. $P_p(-\,|\,\mathrm{I-,D-})$:

$$\frac{\mathrm{P_t}(\mathrm{I-,D-})\times[P_p(-\,|\,\mathrm{I+,D-})\times \mathrm{P_t}(\mathrm{I+,D-})+P_p(-\,|\,\mathrm{I+,D+})\times \mathrm{P_t}(\mathrm{I+,D+})]}{[P_p(-\,|\,\mathrm{I+,D-})\times \mathrm{P_t}(\mathrm{I+,D-})+P_p(-\,|\,\mathrm{I+,D+})\times \mathrm{P_t}(\mathrm{I+,D+})+P_p(-\,|\,\mathrm{I-,D+})\times \mathrm{P_t}(\mathrm{I-,D+})+P_p(-\,|\,\mathrm{I-,D-})\times \mathrm{P_t}(\mathrm{I-,D-})]^2},\ p=1,2,3$$

w.r.t. $P_p(-\,|\,\mathrm{I-,D+})$:

$$\frac{\mathrm{P_t}(\mathrm{I-,D+})\times[P_p(-\,|\,\mathrm{I+,D-})\times \mathrm{P_t}(\mathrm{I+,D-})+P_p(-\,|\,\mathrm{I+,D+})\times \mathrm{P_t}(\mathrm{I+,D+})]}{[P_p(-\,|\,\mathrm{I+,D-})\times \mathrm{P_t}(\mathrm{I+,D-})+P_p(-\,|\,\mathrm{I+,D+})\times \mathrm{P_t}(\mathrm{I+,D+})+P_p(-\,|\,\mathrm{I-,D+})\times \mathrm{P_t}(\mathrm{I-,D+})+P_p(-\,|\,\mathrm{I-,D-})\times \mathrm{P_t}(\mathrm{I-,D-})]^2},\ p=1,2,3$$

w.r.t. $P_t(\mathrm{I+,D-})$:

$$\frac{(-1)\times P_p(-\,|\,\mathrm{I+,D-})\times[P_p(-\,|\,\mathrm{I-,D-})\times \mathrm{P_t}(\mathrm{I-,D-})+P_p(-\,|\,\mathrm{I-,D+})\times \mathrm{P_t}(\mathrm{I-,D+})]}{[P_p(-\,|\,\mathrm{I+,D-})\times \mathrm{P_t}(\mathrm{I+,D-})+P_p(-\,|\,\mathrm{I+,D+})\times \mathrm{P_t}(\mathrm{I+,D+})+P_p(-\,|\,\mathrm{I-,D+})\times \mathrm{P_t}(\mathrm{I-,D+})+P_p(-\,|\,\mathrm{I-,D-})\times \mathrm{P_t}(\mathrm{I-,D-})]^2},\ t=1,2,3,4;\ p=1,2,3$$

w.r.t. $P_t(\mathrm{I+,D+})$:

$$\frac{(-1)\times P_p(-\mid \mathrm{I+,D+})\times[P_p(-\mid \mathrm{I-,D-})\times \mathrm{P_t}(\mathrm{I-,D-})+P_p(-\mid \mathrm{I-,D+})\times \mathrm{P_t}(\mathrm{I-,D+})]}{[P_p(-\mid \mathrm{I+,D-})\times \mathrm{P_t}(\mathrm{I+,D-})+P_p(-\mid \mathrm{I+,D+})\times \mathrm{P_t}(\mathrm{I+,D+})+P_p(-\mid \mathrm{I-,D+})\times \mathrm{P_t}(\mathrm{I-,D+})+P_p(-\mid \mathrm{I-,D-})\times \mathrm{P_t}(\mathrm{I-,D-})]^2}, t=1,2,3,4;\ p=1,2,3$$

w.r.t. $P_t(\mathrm{I-,D-})$:

$$\frac{P_p(-\mid \mathrm{I-,D-})\times[P_p(-\mid \mathrm{I+,D-})\times \mathrm{P_t}(\mathrm{I+,D-})+P_p(-\mid \mathrm{I+,D+})\times \mathrm{P_t}(\mathrm{I+,D+})]}{[P_p(-\mid \mathrm{I+,D-})\times \mathrm{P_t}(\mathrm{I+,D-})+P_p(-\mid \mathrm{I+,D+})\times \mathrm{P_t}(\mathrm{I+,D+})+P_p(-\mid \mathrm{I-,D+})\times \mathrm{P_t}(\mathrm{I-,D+})+P(-\mid \mathrm{I-,D-})\times \mathrm{P_t}(\mathrm{I-,D-})]^2}, t=1,2,3,4;\ p=1,2,3$$

w.r.t. $P_t(\mathrm{I-,D+})$:

$$\frac{P_p(-\mid \mathrm{I-,D+})\times[P_p(-\mid \mathrm{I+,D-})\times \mathrm{P_t}(\mathrm{I+,D-})+P_p(-\mid \mathrm{I+,D+})\times \mathrm{P_t}(\mathrm{I+,D+})]}{[P_p(-\mid \mathrm{I+,D-})\times \mathrm{P_t}(\mathrm{I+,D-})+P_p(-\mid \mathrm{I+,D+})\times \mathrm{P_t}(\mathrm{I+,D+})+P_p(-\mid \mathrm{I-,D+})\times \mathrm{P_t}(\mathrm{I-,D+})+P_p(-\mid \mathrm{I-,D-})\times \mathrm{P_t}(\mathrm{I-,D-})]^2}, t=1,2,3,4;\ p=1,2,3$$

We provide below the steps for computing the standard errors for the Sensitivity measure for each item and time point. Let us define with $S_{pt}=(S_{pt1,},...,S_{pt4})$ the row vector for item *p* at time *t* with elements the first derivatives of the Sensitivity measure with respect to the four parameters given in the row vector $\vartheta=[P_p(+\mid \mathrm{I+,D-}),\mathrm{P_p}(+\mid \mathrm{I+,D+}),\mathrm{P_t}(\mathrm{I+,D-}),\mathrm{P_t}(\mathrm{I+,D+})]$.

According to the Delta method: $Var(Sensitivity_{pt})=S_{pt}Cov(\vartheta)S_{pt}{}^{T}$

where *p* = 1,2,3 and *t*= 1,2,3,4. Similarly we obtain variances and their corresponding standard errors for the rest of the constructed measures (Specificity, PPV and NPV). We have calculated average values across time for all the measures (Sensitivity, Specificity,

PPV and NPV). Therefore the variance and standard errors of those averages are computed accordingly. In Table 4 in the main article we provide 95 % confidence intervals using standard errors of those averages accordingly.

WEB TABLE 1. Comparison of Alternative Latent Structures of the Single-Level LMMs

	Tanzania Children (N=367)<br>Item response probabilities are assumed to be constant over time						
Model	*J*	*Transition matrices*	*r*	*LL*	*BIC*	*AIC*	*Sample adjusted BIC*
1	2	4	15	-1284.1	2656.8	2598.2	2609.2
2	2	2	11	-1290.3	2645.5	2602.6	2610.6
3	3	4	35	-1224.1	2654.9	2518.2	2543.8
4	3	2	23	-1242.6	**2621.1**	2531.3	2548.1
5	4	4	63	THE BEST LOG-LIKELIHOOD VALUE WAS NOT REPLICATED[a]	NA[b]	NA	NA
6	4	2	39	-1210.766	2651.8	**2499.5**	**2528.1**

	Gambia Children (N= 587)<br>Item response probabilities are assumed to be constant over time						
Model	*J*	*Transition matrices*	*r*	*LL*	*BIC*	*AIC*	*Sample adjusted BIC*
1	2	4	15	-1267.3	2630.3	2564.7	2582.7
2	2	2	11	-1269.7	**2609.6**	2561.5	2574.7
3	3	4	35	-1235.1	2693.2	2540.0	2582.0
4	3	2	23	-1237.4	2621.4	2520.7	2548.3
5	4	4	63	THE BEST LOG-LIKELIHOOD VALUE WAS NOT REPLICATED[a]	NA[b]	NA	NA
6	4	2	39	-1207.552	2663.7	**2493.1**	**2539.9**

Abbreviations: *r*, number of free parameters; *LL*, corresponding maximum log-likelihood; *BIC*, Bayesian Information Criterion; *AIC*, Akaike Information Criterion

Model 1, 3, 5: transition probabilities τ vary during all intervals; *Model 2, 4, 6*: baseline and follow-up τ vary such that two transition probability matrices were fitted (one for 0-2 months and one common matrix for each subsequent transition period).

Tanzania Children (N=367)<br>Item response probabilities are allowed to vary over time							
Model	*J*	*Transition matrices*	*r*	*LL*	*BIC*	*AIC*	*Sample adjusted BIC*
1A	2	4	39	-1220.9	2672.1	2519.7	2548.4
2A	2	2	17	-1253.4	**2607.2**	2540.8	2553.3
3A	3	4	71	-1179.1	2777.5	2500.2	2552.2
4A	3	2	32	-1219.4	2627.7	2502.7	**2526.2**
5A	4	4	111	THE BEST LOG-LIKELIHOOD VALUE WAS NOT REPLICATED	NA	NA	NA
6A	4	2	51	-1194.207	2689.6	**2490.4**	2527.8

Gambia Children (N= 587)<br>Item response probabilities are allowed to vary over time							
Model	*J*	*Transition matrices*	*r*	*LL*	*BIC*	*AIC*	*Sample adjusted BIC*
1A	2	4	39	-1238.5	2725.6	2555.0	2601.8
2A	2	2	17	-1256.1	2620.6	2546.2	2566.6
3A	3	4	71	-1194.9	2842.5	2531.9	2617.1
4A	3	2	32	-1221.2	2646.4	2506.4	2544.9
5A	4	4	111	THE BEST LOG-LIKELIHOOD VALUE WAS NOT REPLICATED	NA	NA	NA
6A	4	2	51	-1200.399	2725.9	2502.8	2564.2

Model 1A, 3A, 5A: ρ and τ vary during all intervals; *Model 2A, 4A, 6A*: Baseline and follow-up τ vary such that two transition probability matrices were fitted (one for 0-2 months and one common matrix for each subsequent transition period).

[a]Because of the multimodal likelihood of LMMs, and perhaps to the large r's and power in the analyzed data, the best log-likelihood value was not replicated. Consequently results of estimated parameters from this model cannot be trusted

[b] Abbreviation NA, Not Available; as the best log-likelihood value was not replicated, results for BIC, AIC and sample adjusted BIC form this model cannot be trusted either

Tables above report the LL, the number of latent health states *k*, parameters *r*, the BIC, AIC and sample adjusted BIC value for the LMMs that were obtained during the model selection.

Highlighted values in bold indicate the lowest obtained information criteria. The *r* is calculated by P_η: number of latent health state prevalences; P_ρ number of item response probabilities and P_τ number of transition probabilities estimated.

The formula for the sample adjusted BIC value is nearly identical to the formula for BIC with the difference that it replaces in the latter *n* with *n** (where n* = (n + 2) / 24). Such information criteria are not fully yet understood in statistical literature and how they particularly function for LMMs. We explored a number of LMMs and determined as the most appropriate the model that combined most of the following principles: reasonable goodness of fit, parsimony through minimum values for most of the information criteria listed above as well as more apparent biological plausibility of the phenomenon studied here.

We decided to keep the item response probabilities identical across times, because the meaning of the latent health states also remains constant over time for these models. Although for Tanzania BIC for Model 4, AIC for Model 6 and sample adjusted BIC for Model 6 were the smallest. The item response probabilities for Model 6 did not yield latent health states that were biologically interpretable. Thus, we selected Model 4 for further testing.

For The Gambia, BIC was the smallest for Model 2; however AIC and sample adjusted BIC were the smallest for Model 6. Because Model 6 yielded biologically interpretable latent health states, we selected Model 6 for further testing.

WEB TABLE 2. Fit Criteria for Nonparametric Multilevel Latent Markov Model (LMM) Specification

Tanzania Children (N=367)							
Model	*r*	*m*	LL	*BIC*	AIC	Sample adjusted BIC	*p-value**
7	22		-1097.3	2330.4	2240.6	2257.4	
8	40	2	-1043.0	**2322.1**	**2185.2**	**2208.6**	<0.001

Gambia Children (N=587)							
Model	*r*	*m*	LL	*BIC*	AIC	Sample adjusted BIC	*p-value**
7	38		-1071.4	2391.5	2220.9	2267.7	
8	52	2	-976.6	**2284.8**	**2057.3**	**2119.7**	<0.001

Remarks: When nonparametric multilevel models were fitted to the 5 timepoints under study for both Tanzania and Gambia, warning messages were obtained about the standard errors of some of the model parameters that they may not be trustworthy most probably as an indication of model nonidentification. Therefore, all models presented in the above table were also restricted to the first 4 timepoints of the studies: baseline, 2, 6 and 12 months. Highlighted values in bold indicate the lowest obtained information criteria.

Abbreviations: *r*, number of free parameters; *m*, number of between-level latent classes at the random coefficients part; *LL*, corresponding maximum log-likelihood*; BIC,* Bayesian Information Criterion; *AIC,* Akaike Information Criterion .

Model 7: Fixed effects LMM with 3 and 4 latent health states for Tanzania and Gambia, respectively; There were two transition probability matrices in each case. *Model 8:* Nonparametric multilevel LMM model with 3 and 4 latent health states for Tanzania and Gambia, respectively and 2 between-level latent classes for both countries; *p-value compares the LL between Models 7 and 8; because for both datasets these are significant, the results all imply that Model 8 provides significantly improved fit over Model 7.

Web Table 1 reports the LL value, the number of parameters *r* and the information criteria for single-level and nonparametric multilevel LMM models. All results reported in main text are estimated from Model 8. Model 7 for Tanzania is the same structure as model 4 in Web Table 2,

except fitted to only data from 4 timepoints and of course for Gambia is the same structure as model 6 in Web Table 2 except fitted to only data from 4 time points. The *r* is calculated as in single-level models by P_η: number of latent health state prevalences; P_ρ number of item response probabilities and P_τ number of transition probabilities estimated. In technical terms of MPLUS and the nonparametric multilevel models fitted here, latent categorical variables $C_{i,t}$ that represent the level-1 health states at each time point *t* were regressed on the new categorical latent variable *Cb* and thus the parameters estimated from these regressions are the additional parameters now contained in Web Table 1 if compared with fixed effects models in Web Table 2.
